# Evidence Synthesis of the Ethical and Legal Challenges of Generative Artificial Intelligence in the Provision of Patient Health Information

**DOI:** 10.1049/htl2.70102

**Published:** 2026-10-14

**Authors:** Krithika Anil, Inocencio Daniel Cortes Maramba, David Paul Cook, Daniel Cyrus, Zhamayne Fakharuzi, Amir Hossein Namdar, Timothy Owen West, Sandeep Shirgill

**Affiliations:** ^1^ Faculty of Health University of Plymouth Plymouth, England UK; ^2^ University of Plymouth Plymouth, England UK; ^3^ E‐Mind Ltd Bakewell, England UK; ^4^ Department of Computer Science, Faculty of Engineering and Physical Sciences University of Surrey Guildford, England UK; ^5^ Kingston University Research and Impact London UK; ^6^ Department of Mathematics, School of Natural Sciences, Faculty of Science and Engineering The University of Manchester Manchester, England UK; ^7^ Department of Clinical and Movement Neurosciences University College London London, England UK; ^8^ Department of Immunology and Immunotherapy, College of Medicine and Health University of Birmingham Birmingham, England UK; ^9^ School of Mathematics, College of Engineering and Physical Sciences University of Birmingham Birmingham, England UK

**Keywords:** AI, artificial intelligence, chronic illness, health information, healthcare, large language models, long‐term health conditions

## Abstract

Large language model (LLM)‐based generative AI offers the promise of responsive health information for patients and carers but presents ethical and legal challenges when used outside clinical oversight. This review maps these considerations for patients accessing information on long‐term conditions in non‐clinical settings. Following PRISMA guidelines, we searched databases (MEDLINE, CINAHL, EMBASE, and Web of Science) between April and May 2025. The review synthesises 24 cross‐sectional studies regarding patient‐facing LLMs, excluding clinician‐support tools. Results indicate that LLMs perform well on general topics but struggle with specialised information, often generating complex responses with unreliable citations. Ethical concerns highlight inaccuracy, insufficient empathy, and the potential exacerbation of health inequalities, while analyses of legal challenges focus mainly on liability and consent. We conclude that current technical limitations and regulatory gaps regarding device classification and safety obligations could pose risks to patients. Consequently, stakeholders must establish clear accountability frameworks, and LLMs should currently function only as supplementary tools rather than replacements for expert clinical advice. Further research into the use of agent‐based LLM architectures, where specialised LLM agents collaborate to verify information, reason symbolically, and interface with patient health records under strict data governance, may provide the solution to the current limitations of LLMs.

## Graphical abstract

This mini‐review examines the ethical and legal challenges of using large language model (LLM)‐based generative AI to provide health information to patients with long‐term conditions in non‐clinical settings. Findings indicate that while LLMs show promise for general health information, their limitations regarding accuracy, readability, and source credibility raise significant ethical and legal concerns, particularly around liability and safety obligations. Consequently, LLMs should currently serve only as supplementary tools, not replacements for expert clinical advice, until clear accountability frameworks and accurate clinical information can be ensured.

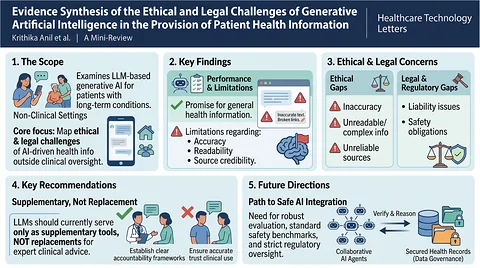

## 1 Introduction

Long‐term conditions, such as stroke or diabetes, require ongoing management outside of clinical settings. Patients and their carers may be overwhelmed by the amount of information they receive about their health condition [1, 2], or they may feel frustrated at the lack of personalised health information relevant to their lives [3]. In recent years, large language model (LLM)‐based generative artificial intelligence (AI) has emerged as a tool with the potential to provide health information to patients and carers in ways that are more responsive and personalised than traditional information sources [4].

LLMs providing health information raise complex ethical and legal challenges when used to deliver personalised health information outside formal clinical oversight, such as potential for inaccurate information, breaches of data privacy, and unclear liability for potential harms. These risks may be amplified in contexts where patients or carers rely on AI instead of or in addition to professional advice without clinical gatekeeping. Addressing these risks by developing LLMs to provide safe and controlled information may lead to improved access to health information for patients and carers.

This is particularly urgent to address because the general public are increasingly using LLMs across all aspects of their lives [5], especially relating to seeking personalised health information where LLMs are described as their personal therapist or doctor [6]. There is also a growing recognition by international bodies of both the opportunities and risks of AI in health. For example, the World Health Organization (WHO) published ethical and governance guidance on large multi‐modal models (LMMs) [7] and states that humans should remain in control of healthcare systems and that AI should be used under appropriate conditions that foster accountability. This is also reflected in the FUTURE‐AI consortium's guidelines for trustworthy AI in health care, which outline six guiding principles: fairness, universality, traceability, usability, robustness, and explainability [8]. However, the practical application of this guidance is unclear, especially for the use of LLMs directly by patients and carers in understanding their health condition. A core regulatory aspect is the intended purpose. Where a patient‐facing LLM is intended to inform diagnosis, prediction, prevention, monitoring or treatment decisions, it is likely to qualify as medical device software. Additionally, LLMs intended for general health information may fall outside medical‐device law but remain subject to data‐protection and consumer obligations.

Recent literature has begun to map the ethical concerns around LLM‐based generative AI in healthcare. For example, recent systematic reviews identified that information accuracy and bias were the most explicit ethical principles discussed [9], yet they conclude that current regulatory and ethical frameworks struggle to keep pace with rapid LLM advancements [10]. Evidence suggests that LLMs should be used in an assistive role rather than an autonomous role that requires robust oversight and safeguards [11].

Despite this growing literature base, there are important gaps that are yet to be addressed. Current literature focuses on general health conditions or clinical settings and decision‐making. Little is known about the ethical and legal use of LLMs outside of clinical oversight, where patients and carers directly engage with the LLM to know more about their health condition. Furthermore, there is little knowledge specifically within populations with long‐term conditions that require lifelong management. In this evidence synthesis, we aimed to map the ethical and legal considerations associated with the use of LLM‐based generative AI that provides personalised health information to patients and carers for long‐term conditions in nonclinical settings. Our objectives were to (1) understand the ethical and legal considerations for the implementation of LLMs reported by current literature, (2) identify the methodological approaches used to assess LLMs’ performance in their information provision, (3) summarise the outcomes of LLM performances, and (4) understand the knowledge gaps in the use of LLMs for health information provision as reported by current literature.

## 2 Methods

This evidence synthesis was conducted as a scoping review and was thus reported following the guidelines described by the Preferred Reporting Items for Systematic Reviews and Meta‐Analyses extension for Scoping Reviews (PRISMA‐ScR) [12].

### 2.1 Eligibility Criteria

The review question was framed according to the Population Concept Context (PCC) framework.

### 2.2 Population

Our target population was people with non‐communicable, long‐term conditions. Management of non‐communicable conditions typically occurs outside of clinical settings compared to those with communicable conditions, resulting in differences regarding ethical and legal considerations when it comes to the use of LLM‐based tools. This restriction also allowed us to focus the review within an increasingly broad and diverse body of literature on the use of LLM‐based generative AI for health information provision.

### 2.3 Concept

We aimed to include studies that contained the use of LLM‐based generative artificial intelligence in providing personalised information to patients and carers via a chat‐based platform. ‘Personalised’ here refers to tailoring of health information to user‐provided prompts and does not relate to diagnostic or treatment recommendations in a clinical setting, as outlined in “context” below. We also aimed to include studies that mentioned ethical and legal considerations of using an LLM for the provision of health information. Studies were excluded if these concepts were not met.

### 2.4 Context

We aimed to include studies that conducted information provision outside of clinical settings. For example, learning more about a health condition or self‐management strategies. Studies were excluded if they were used to support clinicians (e.g., diagnostics, assessments, monitoring, etc.) or used to facilitate health services within a clinical setting.

### 2.5 Types of Literature

All study designs were included. Non‐systematic or non‐scoping literature reviews, opinion pieces, and protocol‐only papers were excluded.

### 2.6 Search Strategy

An initial limited search of PubMed was undertaken to estimate the volume of relevant literature and to identify search terms. A second search using the developed search terms was undertaken and adapted across each of the following databases: MEDLINE, CINAHL, EMBASE, and Web of Science.

Table 1 presents the search terms developed using the PCC framework. Table 2 presents the record of searches. Please note that we initially were interested in the stroke population for this review, which would have informed our larger project on stroke and LLMs. However, we found no relevant papers on stroke. Thus, we expanded our search to the general patient population. No date limit was used.

**TABLE 1:** Search terms developed using the PCC framework.

Population	Concept 1	Concept 2	Concept 3
Stroke	Ethic*	Large language model*	Chat
Patient*	Ethos	LLM	
	Legal	Artificial intelligence	
	Moral	AI	

**TABLE 2:** Record of searches.

ID	Search Term(s)	Hits	Database	Date
1	(stroke OR patient*) AND (ethic* OR ethos OR legal OR moral) AND (large language model* OR LLM OR artificial intelligence OR AI) AND (chat)	73	MEDLINE	29/04/25
2	(stroke OR patient*) AND (ethic* OR ethos OR legal OR moral) AND (large language model* OR LLM OR artificial intelligence OR AI) AND (chat)	6	CINAHL	29/04/25
3	(stroke OR patient*) AND (ethic* OR ethos OR legal OR moral) AND (large language model* OR LLM OR artificial intelligence OR AI) AND (chat)	58	EMBASE	29/04/25
4	(stroke OR patient*) AND (ethic* OR ethos OR legal OR moral) AND (large language model* OR LLM OR artificial intelligence OR AI) AND (chat)	54	Web of Science	29/04/25
5	(((chat) AND (patient)) AND (Artificial Intelligence)) AND (large language model) Sort by: Most Recent ‘chat’[All Fields] AND (‘patient s’[All Fields] OR ‘patients’[MeSH Terms] OR ‘patients’[All Fields] OR ‘patient’[All Fields] OR ‘patients s’[All Fields]) AND (‘artificial intelligence’[MeSH Terms] OR (‘artificial’[All Fields] AND ‘intelligence’[All Fields]) OR ‘artificial intelligence’[All Fields]) AND (‘large language models’[MeSH Terms] OR (‘large’[All Fields] AND ‘language’[All Fields] AND ‘models’[All Fields]) OR ‘large language models’[All Fields] OR (‘large’[All Fields] AND ‘language’[All Fields] AND ‘model’[All Fields]) OR ‘large language model’[All Fields])	129	PubMed	29/04/25

### 2.7 Study Selection

Following the search, all identified references were imported into EndNote (software allowing easy organisation of study references, including study titles and abstracts; manufactured by Clarivate). After removal of duplications, references were uploaded to Rayyan, an online organisational tool for systematic or scoping reviews. Titles and abstracts were screened independently by KA and IM for assessment against the inclusion criteria. KA and IM resolved any conflicts through discussion after this independent review was completed. The full texts of potentially eligible studies were retrieved and assessed in detail against the inclusion criteria by KA and IM. Full text studies that did not meet the inclusion criteria were excluded and were displayed in a PRISMA flowchart (Figure 1). Any disagreements between reviewers were resolved through discussion by KA and IM, and the rest of the co‐authors checked this review process iteratively to reduce the risk of researcher bias.

**FIGURE 1:**
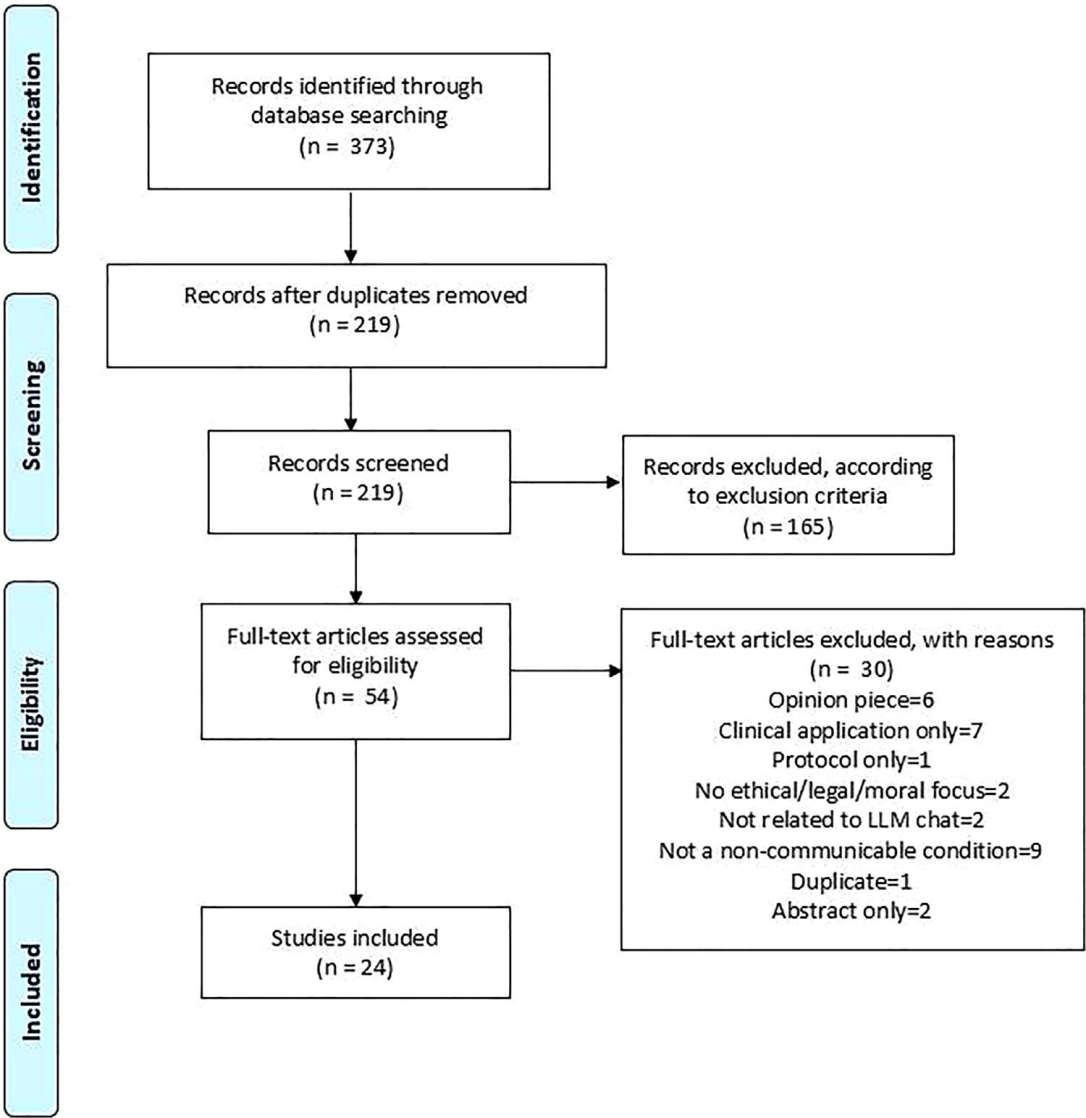
PRISMA flowchart of the review process.

### 2.8 Data Extraction and Synthesis

Data extraction of included studies was conducted by KA and IM. Narrative data extraction was conducted using an Excel spreadsheet according to the objectives of this review. Any disagreements between reviewers were resolved through discussion. The narrative synthesis of the findings was structured according to the review objectives.

## 3 Results

Out of 373 search results, 24 studies were included in the final review (Figure 1; Table 3). All studies were cross‐sectional case studies that evaluated the accuracy of LLM responses, and the majority were from the USA [13, 14, 15, 16, 17, 18, 19, 20, 21, 22, 23, 24, 25] (*n* = 13; 54%).

**TABLE 3:** Characteristics of included studies.

Authors	Country	Aim	Health condition
Clerici et al. (2024) [26]	Italy	To evaluate the quality of the information made available by the chatbot programmes queried about rhabdomyosarcoma in terms of the description of the disease, its treatment, and its prognosis.	Rhabdomyosarcoma
Anees et al. (2025) [27]	Pakistan	To address a critical gap in understanding the accuracy of AI‐generated health information by examining the quality of information provided by ChatGPT.	Varicose veins
Bains et al. (2024) [13]	USA	To compare: (1) orthopaedic surgeons’ evaluation of the appropriateness of the answers to the most frequently asked patient questions after total knee arthroplasty, and (2) patients’ comfort level in answering their post‐operative questions by using answers provided by arthroplasty‐trained nurses and ChatGPT.	Total knee arthroplasty
Cankurtaran et al. (2023) [28]	Turkey	To evaluate the performance of ChatGPT within the context of inflammatory bowel disease, which is expected to become an increasingly significant health issue in the future. In addition, the objective of the study was to assess whether ChatGPT serves as a reliable and useful resource for both patients and healthcare professionals.	Inflammatory bowel disease
Carlson et al. (2024) [14]	USA	To elucidate the accuracy and readability of AI chatbots in answering patient questions regarding urology.	Vasectomy
Gravina et al. (2024) [29]	Italy	To examine ChatGPT's potential to provide medical information regarding questions commonly addressed by patients with inflammatory bowel disease to their gastroenterologists.	Inflammatory bowel disease
Grilo et al. (2025) [30]	Portugal	To evaluate the quality and reliability of ChatGPT's responses to common patient queries about radiotherapy, comparing the performance of ChatGPT's two versions: GPT‐3.5 and GPT‐4.	Cancer
Hermann et al. (2023) [15]	USA	To quantify the accuracy of ChatGPT in answering commonly asked questions pertaining to cervical cancer prevention, diagnosis, treatment, and survivorship/quality‐of‐life.	Cervical cancer
Kozaily et al. (2024) [16]	USA	To investigate the potential of an LLM‐based AI chat platforms in complementing the delivery of patient‐centred care.	Heart failure
Lee et al. (2024) [31]	Republic of Korea	To develop a healthcare chatbot service (AI‐guided bot) that conducts real‐time conversations using large language models to provide accurate health information to patients.	Cancer
Lockie et al. (2024) [32]	Australia	This study aimed to evaluate a ChatGPT generated patient information leaflet against a surgeon generated version in order to explore a potential application of this artificial intelligence language processing model.	Laparoscopic cholecystectomy
Nguyen et al. (2024) [17]	USA	To identify the highest overall performing LLM chatbot for answering FAQs related to anaesthesia.	Anaesthesia
Pirkle et al. (2025) [18]	USA	To assess the reliability of machine learning tools in providing appropriate recommendations for the care of common paediatric orthopaedic conditions.	Paediatric orthopaedic conditions
Sarangi et al. (2023) [33]	India	To assess the capability of ChatGPT‐3.5 in simplifying radiological reports to facilitate improved understanding by healthcare professionals and patients.	Radiology
Sezgin et al. (2025) [19]	USA	To assess AI‐supported (LLM‐supported) tools in providing useful and reliable information to carers of children with cancer.	Cancer
Shen et al. (2024) [20]	USA	To analyse the readability, understandability, and accuracy of ChatGPT‐3.5 responses to a spectrum of user‐generated patient queries and compare them to results from traditional web searches.	Otolaryngology
Steimetz et al. (2024) [21]	USA	To investigate the ability of chatbots to accurately simplify anatomic pathology reports for patients and identify key elements of the reports that are pertinent to patient care.	Pathology
Tailor et al. (2024) [22]	USA	To determine the appropriateness of ophthalmology recommendations from an online chat‐based AI model to ophthalmology questions.	Ophthalmology
Toiv et al. (2024) [23]	USA	To assess the suitability of ChatGPT responses to questions on a relevant medical topic that has an inherently high level of biological complexity and challenging technical terminology.	Gastroenterology
Varady et al. (2024) [24]	USA	To evaluate the ability of ChatGPT‐4 to respond to patient inquiries concerning ulnar collateral ligament injuries and compare these results with the performance of Google.	Ulnar collateral ligament injuries
Vassis et al. (2024) [34]	Denmark	To evaluate the ability of ChatGPT to generate educational information for orthodontic patients based on quantitative data.	Orthodontics
Yang et al. (2024) [25]	USA	To compare the responses of ChatGPT and Bard regarding treatments for hip and knee osteoarthritis with the American Academy of Orthopaedic Surgeons Evidence‐Based Clinical Practice Guidelines recommendations.	Osteoarthritis
Zeljkovic et al. (2025) [35]	Croatia	To assess ChatGPT‐4's ability to inform patients about atrial fibrillation, covering a wide range of questions from understanding the condition to optimising care	Atrial fibrillation
Zhang et al. (2024) [36]	Singapore	To evaluate the accuracy and relevance of ChatGPT's responses to FAQs regarding total knee replacement to assess its clinical utility as a tool for patient education and preoperative decision‐making.	Total knee replacement

Abbreviations: AI, Artificial intelligence; ChatGPT, Chat generative pre‐trained transformer; FAQs, Frequently asked questions; LLM, Large language model; USA, United States of America.

### 3.1 Overview of Included Studies

The study designs primarily comprised cross‐sectional studies [13, 17, 19, 20, 21, 22, 23, 24, 25, 27, 32, 33, 34, 35, 36] and single case/case series designs [14, 15, 16, 18, 26, 28, 29, 30, 31]. The sample sizes of human evaluators or patient participants varied widely, ranging from studies with no explicitly stated sample size for evaluators [18, 29] or with a minimum of two expert reviewers [15, 20, 24, 25, 28, 36] to larger cohorts of patients or physicians, such as 43 participants [32], 50 non‐medical experts [31], 74 individuals [34], and up to 258 patients [13]. Most studies (*n* = 13) utilised small panels of experts, typically between 2 and 5 individuals, for evaluation [14, 15, 16, 17, 19, 20, 21, 23, 24, 26, 27, 28, 36].

LLMs investigated were predominantly various versions of ChatGPT (including ChatGPT‐3, ChatGPT‐3.4, ChatGPT‐3.5, and GPT‐4), which was assessed in all 24 included studies. Other LLMs also evaluated included Google Bard/Gemini [14, 16, 17, 18, 19, 21, 25, 26, 34], Microsoft Bing Chat/Copilot [14, 17, 19, 26], Perplexity AI [14], Anthropic Claude [14], and Google SGE [19].

A broad spectrum of medical conditions were investigated, which included oncology (e.g., rhabdomyosarcoma [26], various types of cancer [15, 19, 30, 31], cardiovascular diseases (e.g., heart failure [16], atrial fibrillation [35]), musculoskeletal conditions (e.g., total knee arthroplasty [13], common paediatric orthopaedic conditions [18], ulnar collateral ligament injuries [24], osteoarthritis [25], total knee replacement [36]), and various surgical or medical specialities (e.g., varicose veins [27], inflammatory bowel disease [14, 28], vasectomy [14], laparoscopic cholecystectomy [32], anaesthesia [17], radiology [33], otolaryngology [20], pathology [21], ophthalmology [22], gastroenterology [23], orthodontics [34]).

### 3.2 Methodological Approaches

The studies employed diverse methodological approaches to evaluate LLM‐generated patient information. A common strategy involved generating questions from various sources, including frequently asked questions (FAQs) from reputable medical websites and guidelines [15, 17, 20, 23, 24, 27, 31, 36], patient forums [16, 20], and direct input from clinical experts [18, 19, 26, 28, 29, 35]. Some studies specifically rephrased questions to an accessible reading level, such as the US 8th grade [17].

Evaluation of LLM responses consistently relied on panels of clinical experts across various specialities, including vascular surgeons [27], orthopaedic surgeons [13, 24, 36], gastroenterologists [23, 28], urologists [14], IBD‐expert physicians [29], radiotherapy experts [30], gynaecologic oncologists [15], heart failure cardiologists [16], oncology specialists [19, 31], general surgeons and surgical trainees [32], anaesthesia experts [17], pathologists [21], ophthalmologists [22], orthodontics experts [34], and cardiologists [35]. Many studies ensured blinding of evaluators to the specific LLM model or other evaluators' scores to minimise bias [15, 17, 21, 24, 28, 30].

The following is a list of the key evaluation metrics and how they were examined:

- Accuracy and appropriateness of information were assessed using multi‐point Likert scales [13, 14, 17, 19, 23, 26, 27, 31, 36] and/or categorised into degrees of correctness (e.g., comprehensive and correct, partially correct, entirely inaccurate) [15, 16, 21, 22, 27, 34].
- Readability and understandability were measured using established tools like the Flesch Reading Ease Score and Flesch‐Kincaid Grade Level [14, 17, 19, 20, 21, 23, 30], and sometimes the Patient Education Materials Assessment Tool (PEMAT) [20] or the Simple Measure of Gobbledygook (SMOG) scores [23].
- Comprehensiveness, safety, empathy, and ethical considerations were evaluated through Likert scales or qualitative assessment [17, 19, 23, 26].
- Consistency and reproducibility were assessed by comparing multiple responses to the same query or across different LLM versions [16, 20, 27, 30, 31]. Cosine similarity (a metric that presents the similarity of responses from each participant, where the metric represents each participant response as a vector and quantifies similarity by calculating the cosine of the angle between vectors) was used in one case [30].
- Source credibility and legitimacy were investigated by verifying cited references for accuracy and existence [18, 19, 24, 25]. Detection of ‘hallucinations’ (i.e., generation of false information) and AI disclosure in responses were also examined [19, 21].
- Statistical analyses included tests for inter‐rater reliability (e.g., Cohen's kappa, Fleiss’ kappa, Cronbach's alpha, intraclass correlation coefficient) [13, 18, 25, 26, 27, 28], as well as comparative tests like Kruskal–Wallis, Mann‐Whitney, Wilcoxon signed rank, chi‐square, and t‐tests to evaluate differences in performance between LLMs or categories of questions [13, 14, 16, 17, 19, 25, 26, 28, 30, 35]. Descriptive statistics were commonly used to summarise findings [19, 21, 22, 27, 30, 36].

### 3.3 Outcomes of LLM Performances

The review revealed several consistent outcomes regarding the performance of LLMs in providing health information:

### 3.4 Information Accuracy and Appropriateness

LLMs generally demonstrated a high level of accuracy and appropriateness for common and factual medical information [13, 14, 15, 20, 26, 27, 31, 33, 36]. ChatGPT‐4 tended to outperform earlier versions of itself and other LLMs in terms of quality and accuracy [19, 21, 30, 34]. However, accuracy reduced as the complexity and specificity of the medical condition or question increased. LLMs struggled with providing information on specialised information [26], complex or rare diseases [26, 29], nuanced aspects of treatments [15], and gastrointestinal emergencies [23]. They also occasionally promoted non‐recommended treatments [25]. Concerns about factual errors and the potential for inaccurate information were frequently raised [16, 23, 27, 29].

### 3.5 Readability and Understandability for Patients

A recurring finding was that LLM‐generated responses often exceeded the recommended reading levels for general patient populations, frequently being at a university or higher‐grade level [14, 17, 20, 21, 23, 30]. This suggested that the information might be challenging for the general public to understand. Some studies indicated that certain LLMs, like Bard or Google SGE, might offer better readability (determined through self‐rated responses by participants) compared to others, such as ChatGPT [14, 19, 21]. Improved readability could be achieved through additional prompting [20] or post‐processing [21, 31]. Despite these readability challenges, some patients reported finding AI‐generated information more useful, comprehensive, and less anxiety‐inducing than standard texts provided in clinic leaflets or information booklets [13, 34].

### 3.6 Consistency and Reproducibility

LLM responses generally showed good consistency and reproducibility when the same questions were posed multiple times [16, 20, 27, 30, 31]. This suggests a degree of reliability in generating similar content under identical prompts.

### 3.7 Source Citation and Credibility

A significant concern was the quality and legitimacy of sources cited by LLMs. While some studies reported a higher proportion of academic sources compared to traditional search engines for specific queries [24], other studies found that LLMs often referenced studies with errors, or to non‐existent journals, or provided links to unrelated research; this occurred in at least 33% of cases across included studies [18, 25]. This highlighted a lack of transparency regarding data sources [27].

### 3.8 Patient Perception and Experience

Patient satisfaction levels with AI‐generated information were mixed; while some patients preferred LLM answers, many expressed uncertainty about trusting AI for postoperative questions [13]. Feedback on the emotional tone and empathy of AI responses was also varied, where some suggested that LLM responses were not empathetic enough, while others reported that responses had an appropriate level of empathy [19].

### 3.9 Ethical Considerations

Five themes were generated for ethical considerations: accuracy and reliability, sourcing of information, data privacy, security, and empathy during communication.

### 3.10 Accuracy and Reliability

Accuracy and reliability were the most prominent themes highlighted in the literature and covered inaccurate and outdated information, inconsistent responses, insufficient explanations, lack of personalised responses, and unintended negative impacts of LLMs.

The most critical risk identified is the tendency of LLMs to produce inaccurate and outdated information [13, 14, 17, 19, 21, 22, 25, 27, 29, 31, 32, 36], with a high risk of hallucinations (i.e., fabricated information) [16, 18, 19, 20, 27] delivered with unwarranted confidence [20]. The static nature of LLM training data risks providing information that is no longer clinically relevant or aligned with current medical standards [13, 16, 18, 22, 27, 29, 30, 36]. This limitation is particularly concerning in rapidly evolving areas such as drug approvals, treatment protocols, or public health guidance. LLMs cannot assess the accuracy of the information they provide [16, 27, 36], highlighting a further risk. Although LLMs can cite sources, these sources may be outdated or conflicting with current clinical best practices [18]. Additionally, the creative nature of these models can compromise accuracy in healthcare contexts [30]. The unpredictability of user queries further complicates quality control, increasing the likelihood of harmful hallucinations in high‐risk scenarios [21].

Another risk relates to the insufficient depth of LLM responses. Some outputs were deemed incomplete, low‐quality, or lacking nuance by evaluators [17, 28, 30]. This lack of in‐depth responses compromises patients’ ability to understand how their health condition impacts them personally, including understanding of treatments and relevant risks.

LLMs currently lack the ability to meaningfully personalise health information [23]. Their responses are typically generic and cannot account for individual patient needs, preferences, or medical histories to an acceptable level.

Finally, the cumulative effect of inaccurate, impersonal, or misleading outputs may lead to unintended negative consequences. Inaccurate responses can result in poor decision‐making, delay in seeking professional care, or even physical or psychological harm [17, 22, 27, 31]. Moreover, the increasing reliance on AI for health information has the potential to alter the dynamics of the patient‐clinician relationship [16]. This may weaken trust in healthcare professionals or diminish the perceived value of expert human advice [13, 14, 34].

### 3.11 Sourcing of Information

Another ethical concern was related to how LLMs source their information. This theme highlighted fabricated or incorrect references, the lack of transparency in information sourcing, and the impact of the information sourced.

A major issue was the generation of fabricated or incorrect citations [13, 18]. LLMs were observed to reference articles or books that do not exist (i.e., hallucinated references) or misattribute content to the wrong reference. This risk can create a false sense of legitimacy and mislead patients who attempt to verify information or seek further reading based on these references.

LLMs often generated responses without disclosing references unless specifically requested [22, 27, 30]. This lack of transparency makes it difficult for patients, especially those who are not AI‐literate, to assess the credibility of the advice provided. This limits the ability to verify information or contextualise medical content. It also presents a considerable barrier to informed decision‐making for patients when engaging with unfamiliar or complex health topics.

The quality of the information produced by current LLMs is inherently bound to the quality of the underlying training data, also known as ‘garbage in, garbage out’. Several studies highlighted that biases and inaccuracies present in the source material are perpetuated in the LLM outputs [15, 22]. This is especially the case for information drawn from low‐quality or non‐peer‐reviewed content. Another risk was related to language bias, where sources not written in English are under‐represented in LLM training data [20, 33]. This leads to a higher risk of inaccurate or incomplete information when providing information in other languages.

### 3.12 Data Privacy and Security

Another ethical concern related to the protection of data privacy and security [31]. Although this was a prominent concern, there was little information in the literature regarding LLMs for patient use. The literature emphasised that robust safeguards must be implemented to prevent unauthorised access, misuse, or breaches of data confidentiality; however, articles did not discuss this in‐depth and did not detail how this should be done. The absence of clearly defined data handling protocols raises concerns about compliance with existing standards such as the General Data Protection Regulation (GDPR; an EU regulation that governs the processing of personal data and safeguards individuals’ rights over their information) or equivalent frameworks. These frameworks typically engage special‐category data rules that include certain requirements, for example, explicit consent, a data‐protection impact assessment for high risks, and transparency obligations.

### 3.13 Empathy During Communication

Empathy was highlighted consistently as an important feature of patient communication. Several studies noted that LLM outputs were often impersonal or clinical [15, 17, 32], lacking the ‘human tone’ or sensitivity needed in healthcare interactions [14, 26]. This may negatively impact patients who seek reassurance, compassion, or support during vulnerable moments. This is especially problematic when discussing serious or distressing health information.

However, one study [17] reported that LLMs were capable of generating empathetic responses. The difference between this study and others may be related to the sensitivity and risk related to the topic in question. This study asked general questions related to anaesthesia, while studies that found a lack of empathy asked questions related to potential disability, impact on quality of life, and death.

### 3.14 Health Inequality Risks

Two themes were generated for health inequality risks: socioeconomic background and literacy.

### 3.15 Socioeconomic Background

There are differences between freely available and paid LLMs, which have implications for the quality of health information received by users from different socioeconomic backgrounds. Paid versions of LLMs tend to offer more reliable and nuanced responses, including better comprehension of complex queries and more contextually appropriate outputs [15, 16, 17, 18, 30]. However, access to these paid versions is restricted to those who can afford the subscription fee or have access through institutional affiliations. As a result, individuals from higher socioeconomic backgrounds are more likely to benefit from higher‐quality AI‐generated health information [15, 17]. Additionally, those from higher socioeconomic backgrounds may have better digital infrastructures that allow for better access to LLMs [30], which further exacerbates health inequalities.

### 3.16 Literacy

Different types of literacy concerns were raised in the literature regarding LLMs and health inequalities. AI literacy is the ability to interact effectively with AI systems and is important for users to engage effectively with LLMs. Studies recommended that patients must know how to structure prompts and follow up with clarifying questions in order to receive useful and accurate responses [17, 30, 34, 35]. However, this recommendation may be difficult to follow for patients who do not have access to AI education or for those with cognitive difficulties.

The readability of LLM responses was stated to be a barrier for many users [14, 30]. Although LLMs are designed to deliver complex information in an easy‐read format, the resulting responses may still be a barrier to patients with lower reading comprehension.

The issue of readability is exacerbated by patients with lower health literacy [30], who may struggle to recognise the relevance, accuracy, or implications of the information provided by LLMs. Additionally, if an LLM constitutes a software medical device, usability and human‐factor evidence form part of legal conformity, where clear, comprehensible information for lay users is required.

### 3.17 Legal Considerations

There was limited discussion within the literature regarding the legal considerations of LLM use for patients. Legal considerations must be given more weight, as the EU AI Act has begun to apply in stages since 2025: prohibited practices (Feb 2025) and transparency for certain systems, including those that interact with individuals (Art. 50), apply earlier, while most high‐risk obligations, including those for AI embedded in medical devices [37, 38], apply from 2026–2027.

From the included articles in this review, two brief legal topics were identified. First, there is currently no established malpractice liability for harms resulting from LLM‐generated health information [17, 34]. As LLMs typically operate outside regulated clinical systems, accountability in the event of patient harm remains unclear.

Second, legal compliance regarding informed consent is uncertain. LLMs did not always provide information regarding treatment options, side effects, or related risks, which were identified as necessary for informed consent before deciding on treatment. There are currently no specific legal standards or frameworks for communicating such information in a way that satisfies regulatory requirements relating to informed consent [34]. In UK law ‘informed consent’ governs clinician–patient decision‐making (i.e., Montgomery duties, which refer to UK legal requirement for clinicians to disclose material risks and alternatives to patients when seeking informed consent [39]). For consumer LLMs outside clinical care, the key legal duties are data‐protection transparency and lawful processing, consumer information standards, and product safety/liability. When clinicians use any health technology within care, the Montgomery duty remains with the clinician.

### 3.18 Knowledge Gaps

The included studies collectively identified several critical knowledge gaps and areas for future research to enhance the utility and safety of LLMs in providing patient information.

### 3.19 Improving Accuracy and Reliability

There is a clear need for further refinement of LLMs to correct deficiencies, integrate mechanisms for validating and updating information, and ensure the provision of detailed and up‐to‐date content from reliable sources [15, 16, 23, 27, 28, 29, 31, 35]. Fine‐tuning models on datasets of correct answers to specific tasks could improve accuracy and reduce ‘hallucinations’ [21].

### 3.20 Application in Complex Health Scenarios

Significant improvements in reliability are needed before LLMs can be endorsed for emergent health topics or complex medical enquiries, requiring capabilities to identify, triage, and provide clear information on next steps and urgency based on current evidence‐based guidelines [23, 35].

### 3.21 Enhancing Readability and Patient‐Centricity

Future research should focus on optimising LLM outputs for patient‐friendly language and systematically evaluating model responses to questions posed directly by patients [16, 23]. This also includes investigating how LLMs can comply with legal requirements for informed consent and information provision [34].

### 3.22 Comparative Studies and Model Evaluation

More comparative studies are needed to assess the performance of different LLMs against each other, as well as against traditional information sources or human clinicians [17, 30, 34, 36].

### 3.23 Integration and Personalised Information

Future work should explore the ability of LLMs to provide patient‐specific and personalised information, potentially integrating AI tools with other patient interaction platforms for a holistic approach to care [35, 36]. The cost‐saving implications of AI technology for assisting nurses in postoperative settings also warrant investigation [13].

### 3.24 Role of Medical Professionals and Oversight

Experts emphasised the need for medical providers to be actively involved in the development and oversight of medical/health‐focused LLMs to minimise the risk of inaccurate information [20, 22, 23].

### 3.25 Source Identification and Transparency

Research is required to train LLM platforms to identify appropriate and credible sources for the information provided in LLM responses, which also increases transparency about the data sources used [25, 27].

### 3.26 Longitudinal Patient Impact

Longitudinal studies are essential to understand the long‐term impact of LLM use on patients and carers and how their needs and LLM performance might evolve over time [19]. For example, early information provision may focus on health condition education while later provision may focus on nuanced or more complex, personalised situations. This is especially important to consider if LLMs learn user preferences.

## 4 Discussion

This scoping review revealed several themes across ethical domains regarding the accuracy of information, transparency of sources, and accessibility for different user groups. Legal considerations were minimally addressed and lacked depth. It is important to note that all LLMs used in the studies were general‐purpose LLMs, using both free and paid versions. No study evaluated LLM platforms that were developed specifically for health purposes. The following table (Table 4) summarises our key discussion points and is intended as a synthesis of our suggestions rather than a presentation of the findings.

**TABLE 4:** Summary of key discussion points regarding ethical and legal considerations.

Area	Main issue	Suggested approach for solution
Accuracy and reliability of content	LLMs may provide inaccurate health information without clinical oversight that poses risks to patient safety.	Using pre‐screened and approved material to train new LLMs Use auditable architectures like Retrieval Augmented Generation (RAG) Ensure consistent human oversight.
Ethical implementation and guidance	Implementation of ethical guidance is unclear for LLMs that intend to provide health information directly to patients and carers.	Embed ethical considerations during the design of an LLM using existing frameworks, such as ‘design for values’ Include end‐users and other stakeholders as part of the early development stages.
Health inequalities	Differences in access to advanced AI tools, digital literacy, and socioeconomic factors may worsen health disparities.	Careful integration and support for patients that addresses their specific needs.
Legal considerations	LLMs are being conceptualised as tools that may influence clinical decisions, which falls within the scope of medical device regulation. LLMs must be able to meet requirements for safety, performance, and risk management, which may be challenging due to variable outputs.	New LLM developments are needed to produce consistent outputs if LLMs are to be regulated as a medical device Existing legal regulations be refined to consider the current public use and need for health‐specific LLMs.

### 4.1 Ethical Considerations

The most frequently discussed ethical issue was the accuracy and reliability of AI‐generated content, which is in agreement with current literature [9, 10] and raises concerns about potential inaccurate information and its consequences for patients and carers given the absence of clinical oversight. Solutions to address this specific context of health provision are unclear. The majority of existing guidance refers to the development of general AI tools in healthcare, for clinical applications, or for specific tools such as LMMs. Regardless, all guidance emphasises the importance of ethical implementation of AI tools in healthcare [7, 8]. A suggestion to implement ethical considerations in the design of AI tools is to follow the paradigm ‘design for values’ [40] that is based on embedding values of human dignity, freedom, equality, and solidarity within the AI tool design. It is important to note that ‘embedding values’ here represents a complex conceptual and technical challenge and does not imply explicit rule‐based encoding within LLMs. We instead refer to a combination of approaches including dataset curation and post‐deployment governance and monitoring designed to shape model behaviour in ways that better reflect societal and ethical standards. Additionally, design and development should include end‐users and other stakeholders as part of the early development stages, and the specific functional and non‐functional requirements must be defined clearly [7, 8]. Finally, existing approaches can be used to address this issue, such as only using pre‐screened and approved materials for LLM training or embedding an auditable path from question to response generation, such as Retrieval Augmented Generation (RAGs; a method where an LLM retrieves relevant external information at inference time and uses it to augment its generated response).

Interestingly, poor readability and empathy of LLM responses were also discussed in ethical terms within this review. This is surprising considering that LLMs are designed specifically for effective communication [17]. The emphasis on empathetic communication suggests that LLMs should be developed beyond considerations for content accuracy to include emotional appropriateness, especially in settings where patients or carers may be vulnerable or experiencing distress.

Lastly, the literature noted the potential for LLMs to exacerbate health inequalities. The findings suggest that individuals from lower socioeconomic backgrounds or those with limited health or digital literacy may face additional barriers in accessing or interpreting AI‐generated health information. AI literacy concerns are particularly relevant given that the effectiveness of LLM responses depends on how prompts are phrased. Additionally, there is a mismatch between user expectations and the actual capabilities of these tools. LLMs are statistical language models rather than reasoning or decision‐making systems. However, not all patients can reasonably be expected to achieve this level of understanding. Developing AI literacy is unrealistic for certain populations due to any cognitive difficulties brought on by their health condition or limited access to technology, education, or support systems. This reinforces the need for careful integration and support in health contexts.

### 4.2 Legal Considerations

Legal considerations were briefly mentioned within the literature. However, broader considerations need to be addressed before these concerns can be resolved. Reference to liability and informed consent suggests a shift in how LLMs are being conceptualised, from functioning as sources of general health information, similar to health textbooks, to tools that may influence clinical decisions, which falls within the scope of medical device regulation.

When LLMs are intended to influence clinical decisions, they fall within the scope of existing medical device regulations [41]. Currently, general‐purpose LLMs state that they should not be used for such clinical decision‐making, yet the general public are increasingly using these LLMs for their health decisions. Medical devices are strictly regulated, and existing LLMs likely do not meet these regulations [42]. EU/UK regimes require demonstrable safety and performance with controlled change (i.e., structured, risk‐assessed, and validated system updates rather than ad hoc modifications), and the EU AI Act/Medical Device Regulation interplay contemplates lifecycle management for high‐risk medical AI. Internationally, the FDA's final Predetermined Change Control Plan guidance similarly recognises pre‐authorised, bounded updates for AI‐enabled software. Therefore, the question remains as to how LLMs and current legal regulations will be developed to meet current public use and need for health‐specific LLMs [43].

Additionally, the nature of LLMs challenges one main medical device regulatory principle of producing consistent high‐quality outcomes [44]. Traditional software medical devices are controlled and deterministic, allowing developers to validate specific outputs and behaviours. In contrast, LLMs are probabilistic [45], which means that they may generate different outputs for the same input, and developers may have limited control over every possible response. This variability may make it challenging to assess compliance with standards for safety, reliability, and transparency, which are important for medical device approval. Further LLM developments related to evaluation, validation, and monitoring approaches are therefore needed to support their use in clinical contexts.

Finally, there are legal implications in how users are framed. Referring to users as ‘patients’ or implying a patient‐provider relationship could establish a duty of care or require legal responsibilities typically applied to registered health professionals [46]. Even when LLMs are presented as informational tools, their tone, context, or branding may lead users to interpret their outputs as health advice. This raises concerns around liability, negligence, and potential breaches of professional regulation, especially if the LLM is integrated into or recommended by healthcare services. However, for consumer tools, consumer protection and product liability rules (e.g., CRA 2015 [47]; CPA 1987 [48]) also apply, and liability disclaimers cannot exclude responsibility for death or personal injury caused by negligence.

### 4.3 Methodological Approaches for Evaluation of LLM Performance

The methodological approaches employed across all included studies prioritise clinical safety and accountability. The nearly universal reliance on small panels of clinical experts (typically 2 to 5 individuals) to evaluate content accuracy and appropriateness reflects the necessity of ensuring clinical oversight.

The methodology of blinding evaluators to the specific source of the responses and calculating inter‐rater reliability (e.g., Cohen's kappa and Cronbach's alpha) is accepted by the literature in this review as a robust evaluation method. The evaluation measures also involved usability metrics that addressed the patient experience, highlighting an understanding that provision of health information must also be accessible. This methodological emphasis directly relates to the ethical principle of universality outlined in guidelines for trustworthy AI in health care [7, 8, 40], ensuring that the output is not inherently exclusionary for populations with varying levels of health literacy.

### 4.4 LLM Performance in Health Information Provision

The performance outcomes reveal mixed insight into LLM capabilities. LLMs, especially newer versions, demonstrated an acceptable ability to handle common, factual health questions. This suggests utility for basic informational tasks. However, outcomes also revealed considerable limitations that compromise safety and usability.

Accuracy rapidly diminished when addressing complex or rare diseases, nuanced aspects of treatment, or specific health/medical guidelines. Furthermore, general‐purpose LLMs were found to occasionally promote non‐recommended treatments and struggle with domain‐specific content. This reinforces the conclusion that LLMs should remain in an assistive role rather than an autonomous one, requiring robust technical and clinical oversight. Meskó and Topol [49] argue that such oversight should ensure patients can use LLMs without causing harm, which can be accomplished through comprehensive testing using robust frameworks, as showcased by Bhimani et al.’s [50] LLM evaluation framework. Their evaluation framework was implemented in real‐world use of LLMs in healthcare and focused on output testing rather than the input data. Their three‐year review showed substantial safety improvements (from 80% to 99%) and eliminated severe harm concerns. Future work examining the clinical impact and patient outcomes from this framework will be valuable to assess LLM usefulness.

### 4.5 Future Research

Future research should move towards longitudinal studies to understand the long‐term impact of LLM use on patients and carers. There is a clear need for more comparative studies to benchmark different LLMs not only against each other but against established sources, such as traditional patient information or human clinicians.

Another key gap is that no study evaluated LLM platforms that were developed specifically for health purposes. The research exclusively focused on general‐purpose LLMs (predominantly variants of ChatGPT). An LLM specific to the health domain it aims to support is needed; this will address current issues with general‐purpose LLMs of low accuracy in domain‐specific content and potentially the need for an LLM that is acceptable as a medical device. Future research must also prioritise methods to reduce health inequalities, including investigating how to simplify prompt structuring for users with low health/AI literacy.

Finally, the limited discussion on legal considerations highlights a key knowledge gap. This area requires urgent exploration to establish clear boundaries of accountability, especially as general‐purpose LLMs are increasingly being used by the public for their own health. Methodologically, regulatory sandboxes (e.g., MHRA's AI Airlock) and lifecycle evaluation envisaged by the AI Act/MDR interplay provide pragmatic routes to test and monitor patient‐facing LLMs under real‐world constraints. Future research should evaluate legal and ethical literature databases to expand on the findings in this review.

### 4.6 Strengths and Limitations

As far as we are aware, this is the first scoping review focused specifically on LLMs providing health information outside of clinical settings for people with long‐term conditions. This review followed a rigorous and transparent process reported using PRISMA‐ScR guidelines and the Population–Concept–Context (PCC) framework. We completed a comprehensive database search across multiple relevant databases with independent article screening to minimise researcher bias.

This review also has some limitations. We conducted this review rapidly (in less than 6 months), which may have resulted in some relevant articles being excluded. However, this rapid process was needed due to the speed of LLM developments, where the review's outcomes can be used to directly inform future LLM development. Additionally, we ensured this review was conducted robustly and transparently in order to minimise the risk of unintentionally excluding relevant articles. We also did not complete a quality or risk‐of‐bias assessment. These assessments are not needed for a scoping review, but we acknowledge that this limits the appraisal of methodological rigour of the included articles.

## 5 Conclusion

This scoping review highlights key ethical, legal, and methodological challenges surrounding the use of LLMs in health information provision. LLMs frequently produce inaccurate information or ‘hallucinations’ that call for robust clinical oversight in their development and implementation. Legal considerations were briefly mentioned, yet they are crucial for the future of LLMs in healthcare. The current regulatory landscape is still adapting to accommodate the nature of these models, particularly regarding how to evaluate and assure performance for systems with open‐ended and context‐sensitive outputs. LLMs are increasingly used by the public to make health‐related decisions, and so the question arises as to how existing regulations can evolve to address both the risks and benefits of AI in health settings.

From a methodological perspective, the studies reviewed focused on ensuring clinical safety and reliability, where they often relied on expert panels for evaluation. These methods are essential, but they are limited in their ability to capture the broader usability and accessibility challenges that may arise when these tools are used by diverse populations, particularly those with low health or AI literacy.

LLMs show promise as a tool for health information provision; however, their integration into healthcare must be carefully managed to ensure ethical, legal, and practical considerations are addressed in order to prevent unintended consequences for both users and healthcare systems.

## Author Contributions

**Krithika Anil**: conceptualisation, data curation, formal analysis, investigation, methodology, project administration, validation, visualisation, writing – original draft, writing – reviewing and editing. **Inocencio Daniel Cortes Maramba**: conceptualisation, data curation, formal analysis, investigation, methodology, validation, visualisation, writing – original draft, writing – reviewing and editing. **David Paul Cook**: conceptualisation, writing – reviewing and editing. **Daniel Cyrus**: conceptualisation, writing – reviewing and editing. **Zhamayne Fakharuzi**: conceptualisation, writing – reviewing and editing. **Amir Hossein Namdar**: conceptualisation, writing – reviewing and editing. **Timothy Owen West**: conceptualisation, writing – reviewing and editing. **Sandeep Shirgill**: conceptualisation, writing – reviewing and editing.

## Funding

IM, TW and DC's salary and research costs were partially supported by the N‐CODE Network+ funded by EPSRC/MRC via grant EP/W035030/1. TW is funded in part by a Royal Academy of Engineering Research Fellowship. The authors received no additional financial support for the research, authorship, and publication of this article.

## Ethics Statement

This work involved no human or animal participants and did not use human tissue or data requiring special permissions. No ethical approvals were needed.

## Conflicts of Interest

Z. F. and D. P. C. are employed by E‐Mind Ltd, which is developing ASKSiD, a stroke‐specific LLM tool. All other authors have no conflicts of interest to declare.

## Data Availability

There is no data available, as this is a scoping review. The search can be replicated by following the steps in the methods.
